# Psychiatric symptoms of Turkish combat-injured non-professional veterans

**DOI:** 10.3402/ejpt.v7.29157

**Published:** 2016-04-01

**Authors:** Berna Güloğlu

**Affiliations:** Department of Counseling and Guidance, Faculty of Educational Sciences, Bahcesehir University, Istanbul, Turkey

**Keywords:** Posttraumatic stress disorders, depression, anxiety, functional impairment, combat, injury, veteran

## Abstract

**Background:**

It is well-known that exposure to combat puts individuals at risk for developing adverse psychological problems, including posttraumatic stress disorder (PTSD), depression, anxiety, and health-related behaviour problems and that the presence of combat-related injury increases the risk for psychopathology. Little is known, however, about the consequences of combat among conscripted soldiers fighting against terrorism in their homeland.

**Objective:**

The main aim of the current study was to examine the prevalence of probable PTSD, severity of PTSD symptoms, depression, anxiety, and PTSD-related functional impairment among Turkish combat-injured, non-professional veterans. In addition, investigated were also the possible differences among the symptoms of PTSD, depression, and anxiety of the veterans by the frequency of current cigarette and alcohol use.

**Methods:**

A total of 366 male veterans were assessed by using a demographic information form, which obtained information about injury status and health behaviours, the Posttraumatic Stress Diagnostic Scale (PDS), and the Brief Symptom Inventory (BSI). Symptom frequency and multivariate analysis of variances (MANOVA) were used to analyse the data.

**Results:**

The prevalence of probable PTSD was 46.7% (171) among Turkish veterans while 16.4% experienced severe depression and 18% experienced severe anxiety. “Upset at reminders (65.8%)” was the most common PTSD symptom. “Responsibilities related to home (48.4%)” was the most frequently reported PTSD-related functional impairment. Results indicated that veterans who smoke more than half a pack per day scored significantly higher in severity of PTSD symptoms, depression, and anxiety. Contrary to expectations, there was no significant difference in symptoms of PTSD, depression, and anxiety related to the frequency of current alcohol use.

**Conclusion:**

Turkish non-professional veterans with physical injuries have serious psychiatric problems an average of 13 years after a combat experience. Psychiatric and psychosocial services to Turkish non-professional veterans are a substantial and ongoing need.

Posttraumatic stress disorder (PTSD) and other psychiatric disorders are well-known consequences of exposure to combat (e.g., Afari et al., 2009; Black et al., 2004; Booth-Kewley et al., 2013; Hoge et al., 2004; Ikın et al., 2004; Thanh, Minh, Wilson, & Slane, 2006). Having been injured in combat raises an individual's vulnerability for the development of PTSD and related psychiatric problems. For example, while 2.5% of non-injured Israeli soldiers screened positive for PTSD, 16.7% of those injured met the criteria for PTSD (Koren, Norman, Cohen, Berman, & Klein, 2005). Among US soldiers who were hospitalised following serious combat injuries, 4.2% of them were diagnosed with probable PTSD and 4.4% were diagnosed with depression after 1 month while 12.2% were diagnosed with PTSD and 8.9% with depression after 4 months, and 12.0% were diagnosed with PTSD and 9.3% with depression after 7 months (Grieger et al., 2006).

It is well-known that PTSD is associated with functional impairment in multiple domains including unstable housing and finance, drug, employment, legal, family/relationships, social functioning, and medical problems (Kang, Mahan, Lee, Magee, & Murphy, 2000; Maguen, Stalnaker, McCaslin, & Litz, 2009; Shea, Vujonovic, Mansfield, Sevin, & Liu, 2010; Thomas et al., 2010). Impairment in functioning may be exacerbated by physical health problems. For example, 17.2% of U.S. Gulf War veterans and 11.6% of non-war veterans reported that impairment in their health restricted them in employment and household functioning (Kang et al., 2000).

Exposure to combat is also associated with poor health behaviours. For example, a relationship between cigarette smoking and anxiety disorders has been reported among the Gulf War veterans (Black et al., 2004). A study of Vietnam combat veterans found that veterans who smoked reported higher levels of PTSD symptoms, depression, and trait anxiety compared with those who did not smoke (Beckham et al., 1995). Another study conducted with Vietnam veterans indicated that 28% of those without PTSD were heavy smokers (≥25 cigarettes daily) whereas 48% of those with PTSD were heavy smokers (Beckham et al., 1997). Veterans may smoke cigarettes as a way of coping mechanism for their psychiatric problems. Numerous studies indicate that substance abuse is associated with exposure to combat and often comorbid with PTSD (Bremner, Southwick, Darnell, & Charney, 1996; Nazarian, Kimerling, & Frayne, 2012; Shipherd, Stafford, & Tanner, 2005). The prevalence of substance abuse among male and female veterans has been reported as 12.5 and 6.2%, respectively, while the prevalence of comorbid PTSD and substance abuse was reported as 8.1 and 3.8%, respectively (Nazarian et al., 2012). In a study of military personnel returning from the Gulf War, 15% reported a current alcohol problem (Shipherd et al., 2005) and that alcohol use was significantly associated with PTSD symptoms clusters of re-experiencing, avoidance, and arousal. At least one study has found that the onset of alcohol and substance abuse was related to the onset of symptoms of PTSD, and increasing use of alcohol and substance abuse led to increased PTSD symptoms (Bremner et al., 1996).

Turkey has been struggling with domestic terrorist attacks for the past 3 decades. A separatist organisation called PKK started terrorist attacks against both military personnel and civilians in 1984 in Turkey. The PKK asserts that they advocate the political and cultural rights and self-determination of the Kurdish citizens in Turkey. From 1984 to 2012, 5,480 military personnel, 594 security personnel, 1,844 civil servants, and 5,557 civilians have lost their lives because of terrorist attacks (Report of Investigation of Human Rights Violation Due to Terror and Violence, 2013). The PKK has been listed as a terrorist organisation by the European Union in 2002 and the United States in 2003 (Cronin et al., 2004). The Turkish government embarked on a counterinsurgency operation against these terrorist attacks. Responsibility of conducting such operations was given to the Turkish Armed Forces (TAF). Alongside professional soldiers, soldiers carrying out their compulsory military service under the TAF are also deployed to these operations.

According to the records of Gulhane Military Hospital (GMH), PTSD emerged in 27.5% of Turkish soldiers who served compulsory military service during the intense period of terrorist attacks. After PTSD, the most common psychiatric disorders were conversion disorders (16.6%), anxiety disorders (13.2%), schizophrenia (8.8%), major depression disorders (6.3%), and adjustment disorders (5.9%) (Reports of Human Rights Foundation of Turkey, 1996). Given the ongoing military operations against terrorist attacks, mental health problems are likely to remain an important concern among those troops. The current study evaluated male veterans with compulsory military service who had experienced physical injury. The study had two aims: (1) to assess the prevalence of probable PTSD, severity of PTSD symptoms , depression, anxiety, and PTSD-related functional impairment in Turkish combat-injured, non-professional veterans; (2) to determine whether frequency of current cigarette and alcohol use was associated with severity of PTSD symptoms, depression, and anxiety.

## Methods

### Participants and procedure

A total of 366 male veterans who had been injured due to combat during compulsory military service within the TAF from 1984 to 2012 participated in the current study.

The data were gathered with the permission of the *Mehmetçik Foundation* which was established in 1982 to provide financial support to veterans who have experienced physical injuries and their family members, and also relatives of soldiers who died during combat. In accordance with Article 53 of the 5434 Law on the Pension Fund of the Turkish Republic (www.mevzuat.adalet.gov.tr/html/5023.html), veterans are grouped into six categories based on the severity of organ loss and the degree of non-functionality in life skills. The more serious organ loss is represented by the lower degree. There were a total of 2808 veterans; 145 of whom had suffered first degree injuries, 118 second degree, 307 third degree, 728 fourth degree, 589 fifth degree and 921 six degree.

A set of 763 questionnaires was posted to the veterans by the foundation with a pre-addressed stamped return envelope in November 2012. The questionnaires were sent to all veterans, numbering 263, with first- and second-degree injuries, 150 randomly selected veterans with third- and fourth-degree injuries, and 100 randomly selected veterans with fifth- and sixth-degree injuries. An introductory letter, which explained the purpose and the nature of the study, and informed the participants that their responses would be kept confidential, was sent along with the questionnaires. All the envelopes that were collected at the foundation were received at the end of the February 2013. Family members read the questions to those who were illiterate. The survey had a response rate of 49.15%.

### Measures

#### Demographic Information Form

Participants were asked their age, marital status, and level of education. They were also asked about the date of physical injury and the severity of injury. Current cigarette and alcohol use was assessed with two questions: (1) How often do you smoke in a day? (do not smoke, 1–10 cigarettes a day/11–20 cigarettes a day/over 20 cigarettes a day?), and (2) How often do you use alcohol? (do not drink, at least two or three times a month/at least two or three times a week/every day).

#### Posttraumatic Stress Diagnostic Scale

A 17-item self-report instrument designed to measure the severity of PTSD symptoms (re-experiencing, avoidance, and hyperarousal) related to a single identified traumatic event (Foa, Riggs, Dancu, & Rothbaum, 1993). Participants were asked to rate the severity of the symptom from 0 (never or just one time) to 3 (five times a week/almost every day).

The score range from 0 to 51 reflects the frequency of the PTSD symptoms. There are cut-off scores for categorising severity of symptoms; a score of 10 or less identified as mild, a score between 11 and 20 identified as moderate, a score between 21 and 35 identified as moderate to severe, and a score of 36 or more identified as severe. A diagnosis of PTSD is made only if all the six DSM–IV criteria are endorsed. If one or more criteria are not met, a diagnosis of PTSD is not made. Level of PTSD-related functional impairment was rated in nine areas of life (job, home, friends, entertainment, family, school, sex life, life satisfaction, and all of the above areas.) that were affected after combat experience as a yes or no. The reliability and validity studies of the Turkish version of Posttraumatic Stress Diagnostic Scale (PDS) were conducted by Isikli and Duru (2006) and Aydın, Barut, Kalafat, Boysan, and Beşiroğlu (2012). The internal consistencies of the scale were found as 0.93 in the first study (Isikli & Duru, 2006) 0.90 for total score, 0.81 for re-experiencing subscale, 0.72 for avoidance subscale, and 0.81 for hyperarousal subscale in the second study (Aydın et al., 2012). Cronbach's alpha coefficient for the current study was 0.93 for total scale, and 0.88, 0.80, and 0.87 for subscales of re-experiencing, avoidance, and hyperarousal, respectively.

#### Brief Symptom Inventory

Respondents were asked to respond to 53 items based on the intensity of distress on a 5-point scale ranging from 0 (not at all) to 4 (extremely) considering the past week. The total score range was from 0 to 212, higher scores demonstrating higher probability of psychological symptoms (Derogatis, 1992). Four different studies that were carried out for the adaptation of the scale to Turkish culture reported Cronbach's alpha coefficient as between 0.93 and 0.96 for total score; the values for the subscales were between 0.55 and 0.86 (Sahin & Durak, 1994). Although the original version consists of nine symptom dimensions, factor analysis of the Turkish version revealed five factors, namely anxiety, depression, negative self, somatisation, and hostility. Depression and anxiety subscales were used in the current study. In the present study, Cronbach's alpha coefficient was 0.92 for anxiety and 0.93 for depression.

### Analytic plan

Frequency and percentage analyses were utilised to determine the prevalence of probable PTSD diagnosis and PTSD symptom clusters, depression, anxiety, and PTSD-related functional impairment in the daily lives of Turkish combat-injured veterans. Moreover, multivariate analysis of variances (MANOVA) was employed to investigate whether the frequency of current cigarette use and frequency of current alcohol use were associated with severity of PSTD as measured by the PDS, as well as depression and anxiety symptoms as measured by the Brief Symptom Inventory (BSI) subscales. Bonferroni correction (Miller, 1991) was applied to avoid type I error and significance level was decreased (α/dependent variable=0.016). Box's M test was conducted to assess the equality of covariance assumption. Analysis was conducted with IBM SPSS Statistics 19.

## Results

### Demographic characteristics of participants

The demographic characteristics of participants are presented in Table 1. The majority of veterans were relatively young with over half of the sample belonging to the 31–40 age group. Veterans were grouped into six categories based on the severity of their injury, with the range of injury varying widely across the six categories. The lower degree represents the more serious injuries. The years following the physical injury ranged between 1 and 29 years (12.08±6.56). The majority had elementary school education or less, were married, and had children.

**Table 1:** Demographic characteristics of participants

Characteristics	*N*	%
Age		
22–30	108	29.6
31–40	199	54.5
41–50	58	15.9
Severity of injury		
First degree	69	18.9
Second degree	53	14.5
Third degree	55	15
Fourth degree	71	19.4
Fifth degree	34	9.3
Sixth degree	81	22.1
Years following physical injury		
20–29 years	53	14.5
10–19 years	199	54.8
1–9 years	112	30.7
Level of education		
Illiterate	5	1.4
Literate	11	3.1
Elementary school	202	56.1
High school	129	35.8
University	13	3.6
Marital status		
Married	301	82.7
Single	55	15.1
Widowed	2	0.5
Divorced	6	1.6
Having children		
Have children	278	76.8
Have no children	84	23.2

### Prevalence of symptoms of PTSD, depression, and anxiety

A total of 171 (46.7%) veterans met the criteria for probable PTSD; 97 (26.5%) veterans showed mild, 166 (45.4%) showed moderate, 75 (20.5%) showed moderate to severe, and 28 (7.7%) showed severe PTSD symptoms. Among Turkish veterans, the prevalence of symptoms of depression was found in 60 (16.4%) veterans, whereas 66 (18%) had symptoms of anxiety. Moreover, 75% of veterans with severe PTSD symptoms, 29.3% with moderate-to-severe PTSD symptoms, and 10.2% with moderate PTSD symptoms met the criteria for depression symptoms. 82.1% of veterans with severe PTSD symptom severity, 28% with moderate to severe PTSD symptom severity, and 12% with moderate PTSD symptom severity screened positive for anxiety symptoms.

The most commonly experienced symptom was in the re-experiencing symptom cluster: upset at reminders (65.8%). The other two most frequently experienced symptoms were in hyperarousal symptom cluster: irritability or anger (65.3%) and difficulty in concentrating (64.8%). Only 5.5% (20) of veterans reported no symptoms. The prevalence of PTSD clusters and endorsement of symptoms is presented in Table 2.

**Table 2:** Prevalence of PTSD symptom endorsements among veterans

	Symptom endorsement^a^	
	
PTSD symptoms	*N*	(%)
No symptoms reported	20	5.5
Re-experiencing^b^	297	81.1
Intrusive memories	229	62.9
Dreams or nightmares	205	56
Flashbacks	167	45.6
Upset at reminders	241	65.8
Physiological reactivity	208	56.8
Avoidance^c^	231	63.1
Avoidance of thoughts or feelings	193	52.7
Avoidance of reminders	194	53
Psychogenic amnesia	136	37.2
Loss of interest	194	53
Detachment	185	50.5
Restricted range of affect	196	53.6
Sense of shortened future	169	46.2
Hyperarousal^d^	271	74.1
Insomnia	212	57.9
Irritability or anger	239	65.3
Difficulty concentrating	237	64.8
Hypervigilance	207	56.6
Being jumpy or easily startled	212	57.9

a Endorsement for each symptom required answering ≤1;

b must be at least 1 of 5 symptoms;

c must be at least 3 of 7 symptoms;

d must be at least 2 of 5 symptoms.

### Prevalence of PTSD-related functional impairment

A total of 282 (77%) veterans reported impairment in at least one area of functioning in their life in the past month; the number of people/percentage with number of areas of impairment are as follows: 34 (9.3%), one area; 45 (12.3%), two areas; 47 (12.8%), three areas; 38 (10.4%), four areas; 24 (6.6%), five areas; 30 (8.2%), six areas; 24 (6.6%), seven areas; and 40 (10.9%), all eight areas.

The most frequently experienced impairment among veterans was found with regard to *responsibilities related to home* (48.4%). According to the PTSD symptom severity, *work* is the most frequently reported impairment among veterans with mild PTSD, *responsibilities related to home* among veterans with moderate and moderate-to-severe PTSD symptoms, and *sex life* among veterans with severe PTSD symptoms. The prevalence of impairments in functioning in different areas is presented in Table 3.

**Table 3:** The frequencies of functional impairments in all veterans and veterans with PTSD symptoms

Impairment areas	All veterans	Mild PTSD symptoms	Moderate PTSD symptoms	Moderate-to-severe PTSD symptoms	Severe PTSD symptoms
Work	44.3	**29.2**	42.6	60.3	75
Responsibilities related to home	**48.4**	22.1	**54.3**	**64.9**	71.4
Relationships with friends	46.2	20.8	52.8	61.6	67.9
Fun and leisure activities	47	26	50.3	60.8	75
School	22.7	10.4	21.6	30.6	57.1
Relationships with family	43.4	21.1	48.4	58.1	64.3
Sex life	30.9	4.2	34.8	41.9	**78.6**
General life satisfaction	46.4	19.8	52.5	60.8	75
All	**82.5**	**3.1**	**18.67**	**22.66**	**50**

The figure in bold represents the most frequently observed functional impairment in all veterans and veterans with PTSD symptoms.

### Current cigarette and alcohol use and symptom severity

Two MANOVAs were conducted, one each for each of the individual independent variables (cigarette smoking and alcohol use). Each MANOVA assesses the relationship between frequency of the behaviour and severity of PTSD symptoms, anxiety, and depression.

#### Current cigarette use

Approximately 63% of the veterans smoked, with 42% reporting smoking one pack or more a day. A 4 (non-smoker, smoking between 1 and 10 cigarettes a day, smoking between 11 and 20 cigarettes a day, smoking over 20 cigarettes a day)×3 (symptoms of PTSD, depression, and anxiety) factorial MANOVA was employed to the scores of PDS and depression and anxiety subscales of BSI. The results of Box's M showed the equity of covariance [Box's *M:* 17.042; *F* (18, 224,448)=0.931, *p*>0.05]. The results of MANOVA revealed the significant main effects of frequency of smoking related to symptoms of PTSD symptoms [Wilks’Λ=0.928, *F* (1, 365)=4.724, *p<*0.016, *η* ^2^=0.038)], symptoms of depression [Wilks’Λ=0.928, *F* (1, 365)=6.708, *p*<0.016, *η* ^2^=0.053)], and symptoms of anxiety [Wilks’Λ=0.928, *F* (1, 365)=5.583, *p*<0.016, *η* ^2^=0.044)]. Descriptive statistics of groups (frequency of current cigarette use) with regard to their symptoms of PTSD, depression, and anxiety level are presented in Table 4.

**Table 4:** Means and standard deviations of PDS and BSI depression subscale and BSI anxiety subscale by frequency of current cigarette use

Dependent variables	Frequency of current cigarette use	*M*	SD	*N*	*P*
PTSD symptoms	Non-smoker	16.382	10.526	137	
	Half pack a day	16.832	10.649	76	
	One pack a day	16.860	10.766	98	0.002
	More than one pack a day	22.421	9.950	55	
	Total	17.510	10.693	366	
Depression	Non-smoker	15.779	10.892	137	
	Half pack a day	15.441	10.189	76	
	One pack a day	19.221	13.000	98	
	More than one pack a day	22.960	11.552	55	0.001
	Total	17.710	11.723	366	
Anxiety	Non-smoker	12.356	9.326	137	
	Half pack a day	12.258	8.536	76	
	One pack a day	14.481	10.601	98	0.001
	More than one pack a day	18.075	9.213	55	
	Total	13.764	9.693	366	

Post-hoc analyses with Bonferroni tests for four groups were conducted to see whether there were significant mean differences between symptoms of PTSD, anxiety, and depression. There was significant mean difference between veterans who smoke more than one pack a day and who do not smoke in terms of symptoms of PTSD. In addition, there was a significant mean difference between veterans who smoke one pack a day and veterans who smoke half a pack per day in terms of symptoms of depression and anxiety. However, there was no significant difference between smoking one pack a day and smoking more than one pack a day.

#### Current alcohol use

Approximately 26% of the sample reported drinking, with 10% falling into the “hazardous drinker” group. For the purpose of examining the frequency of current alcohol use as it might relate to differences in the severity of PTSD, anxiety, and depression symptoms, a 3 (non-drinker, mild drinker, and hazardous drinker)×3 (PTSD symptoms, anxiety, depression) MANOVA was performed. The result of the Box's M test indicated that the assumption of homogeneity of covariance was met for this analysis [Box's *M*: 18.816; *F* (12, 50,644)=1.528, *p*>0.05]. MANOVA results revealed no significant effect of the frequency of current alcohol use on symptoms of PTSD [Wilks’Λ=0.979, *F* (1, 365)=7.183, *p*>0.016, *η* ^2^=0.012)], symptoms of depression [Wilks’Λ=0.979, *F* (1, 365)=2.756, *p*>0.016, *η* ^2^=0.007)], and symptoms of anxiety [Wilks’Λ=0.979, *F* (1, 365)=1.094, *p*>0.016, *η* ^2^=0.002)]. Table 5 presents descriptive statistics of groups (frequency of current alcohol use) with regard to their symptoms of PTSD, depression, and anxiety.

**Table 5:** Means and standard deviations of PDS, BSI depression subscale and BSI anxiety subscale by frequency of current alcohol use

	Frequency of current alcohol use	*M*	SD	*N*	*P*
PTSD symptoms	Non-drinker	16.8145	10.67767	271	
	Mild drinker	19.6490	10.62180	58	
	Hazardous drinker	19.2469	10.55520	37	0.60
	Total	17.5096	10.69288	366	
Depression	Non-drinker	17.1987	11.64219	271	
	Mild drinker	18.3932	12.45480	58	0.82
	Hazardous drinker	20.3810	11.00768	37	
	Total	17.7097	11.72304	366	
Anxiety	Non-drinker	13.5246	9.75522	271	
	Mild drinker	14.3853	10.37184	58	
	Hazardous drinker	14.5436	8.17774	37	0.84
	Total	13.7640	9.69311	366	

## Discussion

One of the purposes of the current study was to assess the prevalence of probable PTSD as well as the severity of PTSD symptoms, depression, and anxiety among Turkish veterans who were physically injured during compulsory military service. The results indicate that 46.7% of veterans met the criteria for probable PTSD. The majority of the sample reported moderate–to-severe PTSD symptoms (67%), with a smaller but substantial proportion experiencing moderate-to-severe depressive symptoms (16.4%) and moderate-to-severe anxiety symptoms (18%). The results are consistent with previous studies that show long-term psychiatric effects following combat in multiple types of symptoms (Solomon & Mikulincer, 2006; Thanh et al., 2006). It is notable that the prevalence rates of psychiatric problems are higher in this study compared with most of the previous studies. For instance, Guloglu and Karairmak (2013) found that 29.6% of Turkish combat-injured veterans had PTSD, and 16.6% had depression comorbid to PTSD. Diagnoses of PTSD, depression, and anxiety found among US soldiers deployed to Iraq were 12.9, 7.9, and 7.9%, respectively (Hoge et al., 2004). Similarly, of the US Marines deployed to Iraq or Afghanistan, 7.28% had anxiety disorder, 5.21% had substance use disorder, and 4.76% had PTSD (Booth-Kewley et al., 2013). Findings of the study by Afari et al. (2009) indicated that the prevalence of PTSD (40%) is similar to the findings of this study; however, prevalence of depression (46%) is higher than in this study.

All of the veterans in the current study were physically injured as a result of being shot at during terrorist attacks or mine explosions. There is a high probability that they may have lost their friends before or after their injury. Hoge et al. (2004) showed a significant relationship between the level of combat exposure, encompassing being shot, handling dead bodies, knowing a person who died, or killing enemies, and the prevalence of PTSD. Therefore, these factors may contribute to the severity of their psychiatric symptoms. The fact that the rate of PTSD and related psychiatric problems in the present study was similar to subgroups experiencing injuries and other aspects of combat trauma contributes to the validity of these findings and highlights the consistency of this pattern of psychopathology.

The results of this study also revealed that the most experienced symptom of PTSD in the re-experiencing symptom cluster is “upset at reminders” (65.8%). Unlike the finding of this study, hypervigilance and being jumpy and easily startled which are part of the hyperarousal symptom cluster were the two most commonly experienced symptoms among 9/11 survivors 2–3 years after the event (DiGrande, Neria, Brackbill, Pulliam, & Galea, 2011). Since the terrorist attacks are still life-threatening in Turkey, combat-injured veterans frequently exposed to reminders of their trauma re-experience the events that injured them. For instance, recently terrorist attacks have increased against security personnel (military personnel and police officers) and deaths are reported every day from the region. Being exposed to this news triggers their memories and they get upset due to the reminders.

The study also found that combat-injured veterans experienced substantial functional impairment. While *responsibilities related to home* was the most experienced impairment in all injured veterans, *sex life* was seen as the most frequently reported impairment in veterans who had severe PTSD. A total of 77% of all veterans reported impairment in at least one area of functioning in their life in the past month; the number of people/percentage with number of areas of impairment are as follows: 34 (9.3%), one area; 45 (12.3%), two areas; 47 (12.8%), three areas; 38 (10.4%), four areas; 24 (6.6%), five areas; 30 (8.2%), six areas; 24 (6.6%), seven areas; and 40 (10.9%), all eight areas.

Another aim of the current study was to investigate the possible differences among symptoms of PTSD, depression, and anxiety of physically injured veterans in terms of the frequency of current cigarette and alcohol use. The findings revealed that there are significant differences in the symptoms of PTSD, depression, and anxiety with regard to the frequency of current cigarette use. Veterans who smoke more than one pack a day demonstrated significantly greater severity for all three symptoms assessed (PTSD, depression, and anxiety) compared with veterans who do not smoke at all or smoke less than half a pack a day. Contrary to expectations, there were no significant differences regarding symptoms of PTSD, depression, and anxiety by the frequency of current alcohol use. While some studies have found no relationship between alcohol use and PTSD symptoms (Gurdil, 2007) and even a lower incidence of alcohol use among patients with PTSD (Dalbudak, 2008), the association between PTSD and alcohol consumption is the more common finding (Capone, McGrath, Reddy, & Shea, 2013; Fetzner, Abrams, & Asmudson, 2013). It is assumed that religion is a predominant factor for Turkish veterans for not drinking or reporting themselves as not drinking since it is forbidden to drink alcohol in Islam. Hence, veterans who usually live in rural areas and have grown up in religious families may not drink alcohol because of their religious beliefs. It is also possible that they kept their drinking habit hidden due to the fear of being excluded from society.

There are certain limitations to the present study. First, this study was limited by the use of self-reported measures of PTSD, depression, and anxiety, despite these instruments being well-validated, adopted to Turkish culture, and used in several studies both in Turkey and abroad. Second, the high prevalence of psychiatric symptoms may be the result of secondary benefits of maintaining psychiatric symptoms which should be taken into consideration in further studies. Third, the time passed since the exposure to combat experience, age of veterans, length of military service, number of traumatic events experienced before and after combat, and severity of injury has not been controlled in this study. These factors may have an impact on the level of psychiatric symptoms. Further studies that would specifically address these issues need to be conducted. Finally, the sample consists of injured non-professional veterans. Therefore, it is impossible to generalise the findings to other populations, such as injured professional soldiers, non-injured non-professional soldiers, and soldiers who do not have combat experience.

In spite of these limitations, the current study makes several contributions. The study identifies the frequent occurrence of PTSD symptoms among Turkish veterans, a trauma-exposed population about which little is known. It also highlights the critical need for continued psychological support and intervention strategies to veterans who were physically injured as a result of life-threatening traumatic experiences. The TAF provides psychological and psychiatric support to their personnel during their military service. Mental health services of professional and non-professional military personnel have been conducted through “Guidance and Counseling Centers” in the military and mental health units in their hospitals. Today, TAF has created 397 guidance and counselling centres, and 763 staff are working in these centres. Of the 763 staff, 626 are psychologists and counsellors and 137 are petty officers (www.tsk.tr/3_basin_yayin_faaliyetleri/3_4_tskdan_haberler/2015/tsk_rehberlikvedanismahizmetleri.html). If soldiers had been injured during compulsory military service, they continue to receive physical and psychiatric support from the military hospitals after they have been honourably discharged. However, these services are usually provided in big cities like Ankara, Istanbul, or Izmir.

It can be difficult for those living in small cities to reach these institutions for getting physical and psychiatric help. Therefore, regional needs should be taken into consideration and institutions that care for all types of problems of veterans should be established. In addition, the substantial amount of smoking is likely to have a negative impact on the physical health of veterans and may elevate the symptoms of their injury. Hence, providing psychological support specific to health behaviours may be of benefit as well. To conclude, it is advisable that the government support the establishment of institutions that will provide psychiatric and psychosocial interventions to veterans who were injured serving under the threat of terrorist attacks all over Turkey.

## References

[CIT0001] Afari N, Harder L.H, Madra N.J, Heppner P.S, Moeller-Bertram T, King C, Baker D.G (2009). PTSD, combat injury, and headache in veterans returning from Iraq/Afghanistan. Headache.

[CIT0002] Aydın A, Barut Y, Kalafat T, Boysan M, Beşiroğlu L (2012). Psychometric properties of the Turkish version of the PTSD Symptom Scale-Self-Report (PSS-SR). Anatolian Journal of Psychiatry.

[CIT0003] Black D.W, Carney C.P, Peloso P.M, Woolson R.F, Schwartz D.A, Voelker M.D, Doebbeling B.N, … (2004). Gulf War veterans with anxiety: Prevalence, comorbidity, and risk factors. Epidemiology.

[CIT0004] Beckham J.C, Kirby A.C, Feldman M.E, Hertzberg M.A, Moore S.D, Crawford A.L, Fairbank J.A, … (1997). Prevalence and correlates of heavy smoking in Vietnam veterans with chronic posttraumatic stress disorder. Addictive Behaviors.

[CIT0005] Beckham J.C, Roddman A.A, Shipley R.H, Hertzberg M.A, Cunha G.H, Kudler H.S, Fairbank J.A, … (1995). Smoking in Vietnam combat veterans with post-traumatic stress disorder. Journal of Traumatic Stress.

[CIT0006] Booth-Kewley S, Schmied E.A, Highfill-McRoy R.M, Larson G.E, Garland C.F, Ziajko L.A (2013). Predictors of psychiatric disorders in combat veterans. BMC Psychiatry.

[CIT0007] Bremner J.D, Southwick S.M, Darnell A, Charney D.S (1996). Chronic PTSD in Vietnam combat veterans: Course of illness and substance abuse. American Journal of Psychiatry.

[CIT0008] Capone C, McGrath A.C, Reddy M.K, Shea M.T (2013). Trauma-related correlates of alcohol use in recently deployed OEF/OIF veterans. Journal of Traumatic Stress.

[CIT0036] Cronin A.K, Aden H, Frost A, Jones B (2004). Foreign Terrorist Organizations: CRS Report to Congress.

[CIT0010] Dalbudak E (2008). The relationship of posttraumatic stress disorder and personality characteristics with quality of life.

[CIT0011] Derogatis L.R (1992). BSI: Administration, scoring, and procedures manual—II.

[CIT0012] DiGrande L, Neria Y, Brackbill R.M, Pulliam P, Galea S (2011). Long-term posttraumatic stress symptoms among 3,271 civilan survivors of the September 11, 2001, terrorist attacks on the World Trade Center. American Journal of Epidemiology.

[CIT0013] Fetzner M.G, Abrams M.P, Asmundson G.J (2013). Symptoms of posttraumatic stress disorder and depression in relation to alcohol-use and alcohol-related problems among Canadian Forces veterans. The Canadian Journal of Psychiatry.

[CIT0014] Foa E.B, Riggs D.S, Dancu C.V, Rothbaum B.O (1993). Reliability and validity of a brief instrument for assessing posttraumatic stress disorder. Journal of Traumatic Stress.

[CIT0015] Grieger T.A, Cozza S.J, Hoge C, Martinez P.E, Engel C.C, Wain H.J (2006). Posttraumatic stress disorder and depression in battle-injured soldiers. American Journal of Psychiatry.

[CIT0016] Guloglu B, Karairmak O (2013). Posttraumatic stress disorder among Turkish veterans of the southeast. Anatolian Journal of Psychiatry.

[CIT0017] Gurdil G (2007). Relationship among traumatic experiences, ways of coping, internal-external locus of control beliefs and hazardous alcohol use in university students.

[CIT0018] Hoge C.W, Castro C.A, Messer S.C, McGurk D, Cotting D.I, Koffman R.L (2004). Combat duty in Iraq and Afghanistan, mental health problems, and barriers to care. The New England Journal of Medicine.

[CIT0019] Ikın J.F, Sım M.R, Creamer M.C, Forbes A.B, McKenzie D.P, Kelsall H.L, Schwarz H, … (2004). War-related psychological stressors and risk of psychological disorders in Australian veterans of the 1991 Gulf War. The British Journal of Psychiatry.

[CIT0020] Isikli S, Duru Ç (2006). Pilot Study. Travma sonrası stress belirtileri olan bireylerde olaya ilişkin dikkat yanlılığı, ayrışma düzeyi ve çalışma belleği uzamı arasındaki ilişkiler.

[CIT0021] Kang H.K, Mahan C.M, Lee K.Y, Magee C.A, Murphy F (2000). Illnesses among United States veterans of the Gulf War: A population-based survey of 30000 veterans. Journal of Occupational and Environmental Medicine.

[CIT0034] Koren D, Norman D, Cohen A, Berman J, Klein E.M (2005). Increased PTSD risk with combat-related injury: A matched comparison study of injured and uninjured soldiers experiencing the same combat events. American Journal of Psychiatry.

[CIT0023] Maguen S, Stalnaker M, McCaslin S, Litz B.T (2009). PTSD subclusters and functional impairment in Kosova Peacekeepers. Military Medicine.

[CIT0024] Miller R.G (1991). Simultaneous statistical inference.

[CIT0025] Nazarian D, Kimerling R, Frayne S.M (2012). Posttraumatic stress disorder, substance use disorders, and medical comorbidity among returning US veterans. Journal of Traumatic Stress.

[CIT0026] (1996). Reports of Human Rights Foundation of Turkey.

[CIT0027] Sahin N.H, Durak A (1994). A study of the Brief Symptom Inventory in Turkish youth. Turkish Journal of Psychology.

[CIT0028] Shea M.T, Vujanovic A.A, Mansfield A.K, Sevin E, Liu F (2010). Posttraumatic stress disorder symptoms and functional impairment among OEF and OIF National Guard and Reserve veterans. Journal of Traumatic Stress.

[CIT0029] Shipherd J.C, Stafford J, Tanner L.R (2005). Predicting alcohol and drug abuse in Persian Gulf War veterans: What role do PTSD symptoms play?. Addictive Behaviours.

[CIT0030] Solomon Z, Mikulincer M (2006). Trajectories of PTSD: A 20-year longitudinal study. American Journal of Psychiatry.

[CIT0031] Thanh D.D, Minh T.T, Wilson J.P, Slane S (2006). Posttraumatic stress disorder among Vietnamese war veterans living in Vietnam.

[CIT0035] Thomas J.L, Wilk J.E, Riviere L.A, McGurk D, Castro C.A, Hoge C.W (2010). Prevalence of mental health problems and functional impairment among active component and national guard soldiers 3 and 12 months following combat in Iraq. Archieves of General Psychiatry.

[CIT0032] Turkish Parliament Human Rights Investigation Commission (2013). Report of Investigation of Human Rights Violation Due to Terror and Violence.

[CIT0033] The regulations about types and degrees of people who become disabled in active duty.

